# Visible-Light-Assisted Photoelectrochemical Biosensing of Uric Acid Using Metal-Free Graphene Oxide Nanoribbons

**DOI:** 10.3390/nano11102693

**Published:** 2021-10-13

**Authors:** Chia-Liang Sun, Cheng-Hsuan Lin, Chia-Heng Kuo, Chia-Wei Huang, Duc Dung Nguyen, Tsu-Chin Chou, Cheng-Ying Chen, Yu-Jen Lu

**Affiliations:** 1 Biomedical Engineering Research Center, Department of Chemical and Materials Engineering, Chang Gung University, Taoyuan City 333323, Taiwan; ilovetaihsi@gmail.com (C.-H.L.); w255400@gmail.com (C.-H.K.); jarvis5020huang@gmail.com (C.-W.H.); 2 Department of Neurosurgery, Linkou Chang Gung Memorial Hospital, Taoyuan City 333423, Taiwan; 3 Center for High Technology Development, Vietnam Academy of Science and Technology, Hanoi 100000, Vietnam; ddnguyen161@gmail.com; 4 Institute of Analytical and Environmental Sciences, National Tsing Hua University, Hsinchu 300044, Taiwan; tsuchin.chou@mx.nthu.edu.tw; 5 Center for Plasma and Thin Film Technologies (CPTFT), Department of Materials Engineering, Ming Chi University of Technology, New Taipei City 243303, Taiwan; cychen0111@mail.mcut.edu.tw

**Keywords:** GONR, MWCNT, uric acid, photoelectrochemical, LED

## Abstract

In this study, we demonstrate the visible-light-assisted photoelectrochemical (PEC) biosensing of uric acid (UA) by using graphene oxide nanoribbons (GONRs) as PEC electrode materials. Specifically, GONRs with controlled properties were synthesized by the microwave-assisted exfoliation of multi-walled carbon nanotubes. For the detection of UA, GONRs were adopted to modify either a screen-printed carbon electrode (SPCE) or a glassy carbon electrode (GCE). Cyclic voltammetry analyses indicated that all Faradaic currents of UA oxidation on GONRs with different unzipping/exfoliating levels on SPCE increased by more than 20.0% under AM 1.5 irradiation. Among these, the GONRs synthesized under a microwave power of 200 W, namely GONR(200 W), exhibited the highest increase in Faradaic current. Notably, the GONR(200 W)/GCE electrodes revealed a remarkable elevation (~40.0%) of the Faradaic current when irradiated by light-emitting diode (LED) light sources under an intensity of illumination of 80 mW/cm^2^. Therefore, it is believed that our GONRs hold great potential for developing a novel platform for PEC biosensing.

## 1. Introduction

In recent years, nanocarbon materials, including graphene nanoribbons (GNRs), have been of great interest to the scientific community and have been widely investigated for various applications [1,2,3,4,5]. Among different research fields, nanocarbon-based electrochemical biosensors have attracted significant attention due to the tremendous demands of our modern society. Conversely, semiconductor materials or earth-abundant catalysts have been studied for their photoelectrochemical (PEC) activities, such as energy conversion and water splitting, including hydrogen evolution reaction and oxygen evolution reaction [6,7,8]. Interestingly, semiconductor electrocatalysts with PEC properties can be exploited as photoelectrodes for PEC biosensors [9,10,11,12,13,14,15,16,17,18,19,20]. For example, Li et al. used TiO_2_/g-C_3_N_4_ in conjunction with dendrimer and alkaline phosphatase for the sensitive detection of protein kinase A activity [9]. Lin et al. combined carbon quantum dots-functionalized MnO_2_ nanosheets with glucose oxidase (GO_x_), labeled as AFB_1_-bovine serum albumin, for monitoring aflatoxin B_1_ [10]. Zhou et al. synthesized reduced graphene oxide/BiFeO_3_ nanohybrids on a magnetic microfluidic device for detection of a prostate-specific antigen [12]. More recently, Sun et al. fabricated a CdS–In_2_S_3_ heterojunction for detecting bleomycin [13]. Zhu et al. prepared ultrathin PtNi nanozyme and benzene-ring doped g-C_3_N_4_ for the ultrasensitive sensing of chloramphenicol [15]. Detailed information on recently developed PEC biosensors that use a variety of photoactive materials and light sources is summarized in Table 1 [9,10,12,13,14,15,16,17,18,19,20].

GNRs exhibit extraordinary properties, such as superlubricity, band structure, magnetism, charge/spin transports, high-capacity energy storage, and topological behavior, which are not present in other nanocarbons [21,22,23,24]. As for electrochemical biosensors, Vukojević et al. developed RuO_2_/GNR-modified screen-printed carbon electrodes to enhance the electrocatalytic biosensing of ethanol and nicotinamide [25]. Tang et al. employed N-GNRs-Fe-MOFs@Au nanocomposites to modify a glassy carbon electrode (GCE) in order to detect Galectin-3, a biomarker for heart failure [26]. Li et al. used nitrogen-doped GNRs and n-C60-PdPt as a dual-type responsive electrochemical immunosensor for the quantitative detection of proprotein convertase subtilisin/kexin type 9 [27]. Feng et al. utilized a combination of gold nanocages and GNRs with large specific surface areas and excellent electrical conductivities to develop a stochastic DNA walker [28]. Liu et al. modified a glassy carbon electrode with a heterostructure of core-shell multi-walled carbon nanotubes at reduced graphene oxide nanoribbons (GONRs) for glutathione detection [29]. Pajooheshpour et al. combined Au-Pt nanoclusters, bovine serum albumin, and GNRs to modify a GCE for the electrochemical detection of diazinon [30]. Although numerous studies have focused on nanocarbon-based biosensors, there is little information available on light sources and their effects on the PEC activities of GONRs. In this context, and based on our previous studies [31,32,33,34,35,36], we herein report visible-light-assisted PEC biosensors for the detection of uric acid (UA) by using GONRs with controllable properties. The PEC sensing activities of GONRs, synthesized under different microwave powers for the detection of UA, are systematically investigated via a solar simulator. Furthermore, the effects of wavelengths and irradiation intensities of light-emitting diode (LED) light sources on the Faradaic currents of UA oxidation are explored. These findings are especially interesting for the rational design of miniature power-saving devices or systems based on the PEC activities of GONRs towards point-of-care testing.

## 2. Experimental Section

### 2.1. Chemicals

MWCNTs (Ctube 120, CNT Co., Ltd., Songdo, Korea) were purchased from a new international vendor in replacement of an old vendor in our earlier publications [31,32,33,34,35,36]. The reason being that the original Mistui company has closed their production line in recent years [37]. All the other reagents were of analytical grade and used without further purification.

### 2.2. Preparation of GONRs

The GONRs and their electrodes were prepared by a focused microwave reactor (CEM-Discover, CEM, Charlotte, NC, USA) and a freeze dryer (FDS-1000, EYELA, Tokyo, Japan), following the similar procedures in our previous papers [31,32,33,34,35,36]. It is worthwhile to mention that the nanoribbon powders were collected and placed for drying in a freeze dryer overnight. The ribbon powders were mixed with water, ethanol, and Nafion to form a suspension. 10 µL of the suspension was dropped onto either a screen-printed carbon electrode or a glassy carbon electrode, followed by vacuum drying, to prepare a working electrode.

### 2.3. Characterization

The TEM (JEM-1230, JEOL, Tokyo, Japan) studies were performed to monitor the morphology evolution at an operating voltage of 100 kV. X-ray photoelectron spectroscopy (XPS) (ESCALAB 250, VG Scientific, Waltham, MA, USA) was used to analyze the changes in composition and bonding. The Raman spectra were characterized by a Raman spectrometer (UniDRON, UniNanoTech, Yongin, Korea), with a laser of 532 nm.

### 2.4. Electrochemical and Photoelectrochemical Measurements

A 3 mm diameter glassy carbon electrode (002012, ALS Co., Inc., Tokyo, Japan), a Ag/AgCl electrode (RE-1S, BAS Inc., Tokyo, Japan), and a platinum wire were used as a working electrode, a reference electrode, and a counter electrode, respectively. Cyclic voltammetry and differential pulsed voltammetry measurements were performed by an electrochemical analyzer (CHI7062E, CH Instruments, Inc., Austin, TX, USA). All solutions were prepared with de-ionized water with a resistivity of 18 MΩ/cm. Either a solar simulator (Newport, SP94022A, Newport, Irvine, CA, USA) or a wavelength-switchable LED (WLS-22-A, Mightex, T.O., Canada) were provided as the light source. For the PEC measurement, the distance between the light source and the working electrode was normally kept as 10 cm. When we set up the light intensity, the absorption of the wall of a quartz beaker and the electrolyte were ignored. Through a computer, we could adjust the current of an LED to change the light intensity. For example, the current of the blue light bulb of an LED is around 300 mA for the light intensity of 80 mW/cm^2^, as determined by a power meter (StarLite, Ophir, Darmstadt, Germany).

## 3. Results and Discussion

### 3.1. Characterization

**Figure 1:**
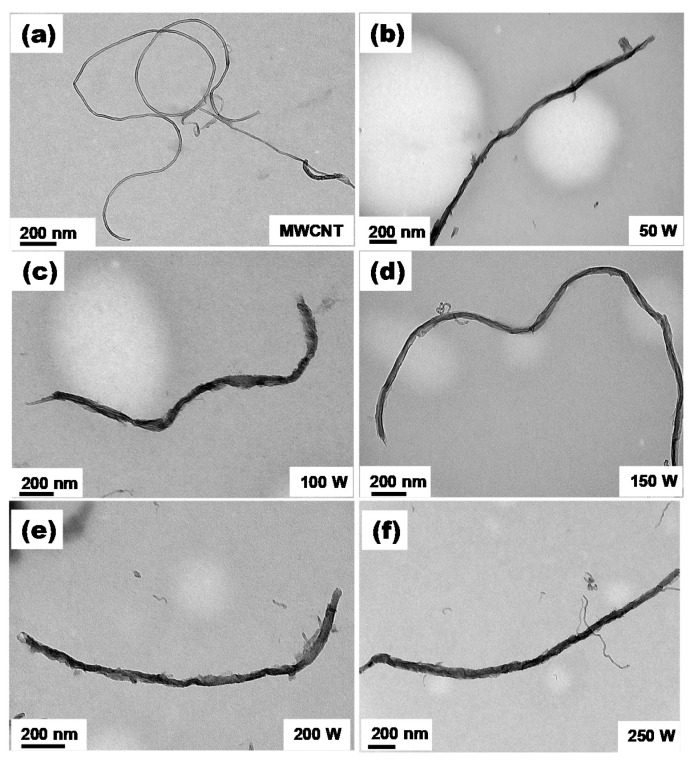
TEM images of (**a**) MWCNT, (**b**) GONR(50 W), (**c**) GONR(100 W), (**d**) GONR(150 W), (**e**) GONR(200 W), and (**f**) GONR(250 W) before and after the microwave-assisted unzipping process at different microwave powers.

Figure 1a displays transmission electron microscopy (TEM) images of the as-purchased/untreated MWCNT, verifying the hollow structure of the material. A microwave-assisted reaction was utilized to unzip the MWCNT in a mixed acid solution. During the reaction, functional groups that contained oxygen could connect to the sidewalls and both ends of the nanotubes, providing attack sites for permanganate ions. Then, sufficient energy input cleaved local carbon–carbon bonds at the outer layers of the nanotubes. Consequently, the nanotubes were gradually unzipped into nanoribbons [31,32]. TEM images of the GONRs unzipped at different microwave powers are shown in Figure 1b–f. The longitudinal length of the GONRs was not considerably different from that of the MWCNTs, demonstrating that the reaction did not shorten the nanotubes. Conversely, the widths of the GONRs were wider than the diameter of the MWCNTs. Compared to other ribbons at higher microwave powers, the sidewalls of GONR(50 W) did not seem to be completely unzipped. Furthermore, the edges of all the ribbon samples were not as continuous as that of our early study, and always contained some broken parts within a certain distance. Figure S1a illustrates the Raman spectra of MWCNTs and GONRs at the excitation laser wavelength of 532 nm. All spectra revealed two main bands, the G band (~1580 cm^−1^) and the D band (~1350 cm^−1^), indicating the characteristics of nanocarbon materials [38,39]. It was found that the I_D_/I_G_ ratios (Figure S1b) of all ribbons were higher than that of MWCNT, indicating the highest value for GONRs unzipped at 50 W. This can be ascribed to the microwave power (50 W) being insufficient for completely unzipping the MWCNTs yet being high enough for a generation of abundant oxygen-containing groups, consequently contributing to the largest amount of defective sites (i.e., highest I_D_/I_G_ ratio). On the contrary, greater powers (100–250 W) could help in unzipping the nanotubes into ribbons at larger extents, thereby possessing more graphitic regions (from the inner walls of the MWCNTs), i.e., contributing more graphitic Raman signals (lower I_D_/I_G_ ratios). These results were in good agreement with the TEM observation, confirming that GONR(50 W) was not entirely unzipped.

**Figure 2:**
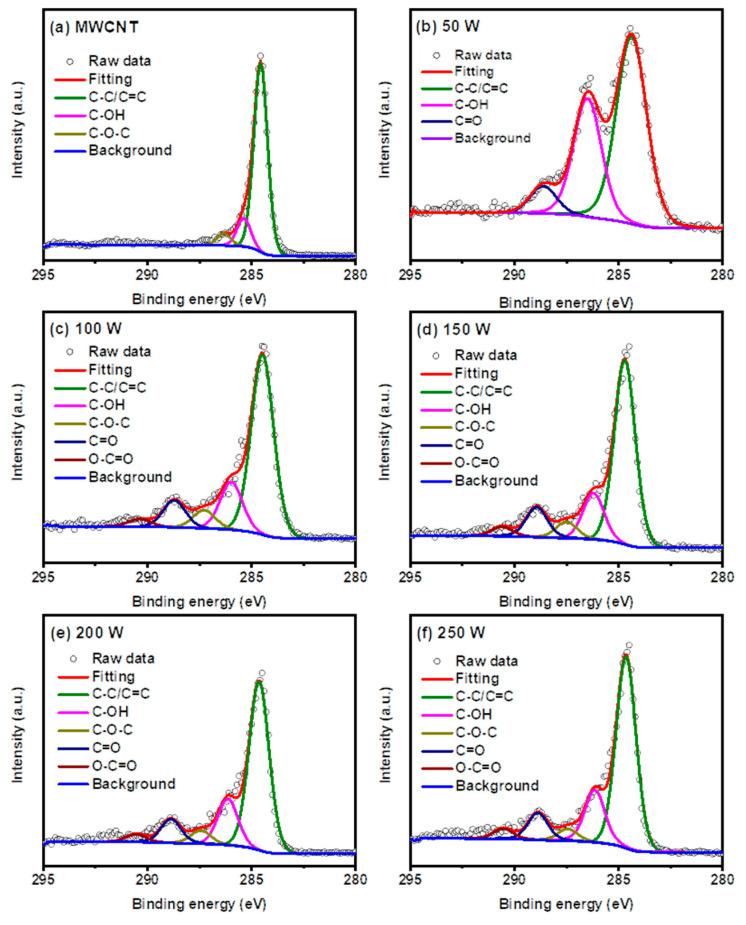
XPS spectra of (**a**) MWCNT, (**b**) GONR(50 W), (**c**) GONR(100 W), (**d**) GONR(150 W), (**e**) GONR(200 W), and (**f**) GONR(250 W) before and after the microwave-assisted unzipping process at different microwave powers.

X-ray photoelectron spectroscopy (XPS) was adopted to determine the surface compositions and the types of functional groups of the MWCNT and the GONRs. Figure 2 shows the XPS C1s spectra of MWCNT and GONR(50 ~ 250 W). The deconvolution peaks belonged to C-C/C=C (284.5 eV), C-OH (286.0 eV), C-O-C (287.3 eV), C=O (288.7 eV), and O-C=O (290.4 eV). The ratios of different functional groups of the MWCNT and the GONRs, derived from Figure 2, are summarized in Figure S2. In Figure S2, it is noticed that the C=O and O-C=O concentrations rose along with increasing microwave powers. Meanwhile, C-OH concentration reached the highest value at 50 W and later declined, implying that the C-OH group might have joined the reaction through the unzipping process. Some C-OH groups remained on the walls without completely unzipping at higher powers. Besides the surface analysis, elemental analyses confirmed the weight ratios of C and O for all samples. The similar trends can be observed.

### 3.2. Electrochemical Measurements

**Figure 3:**
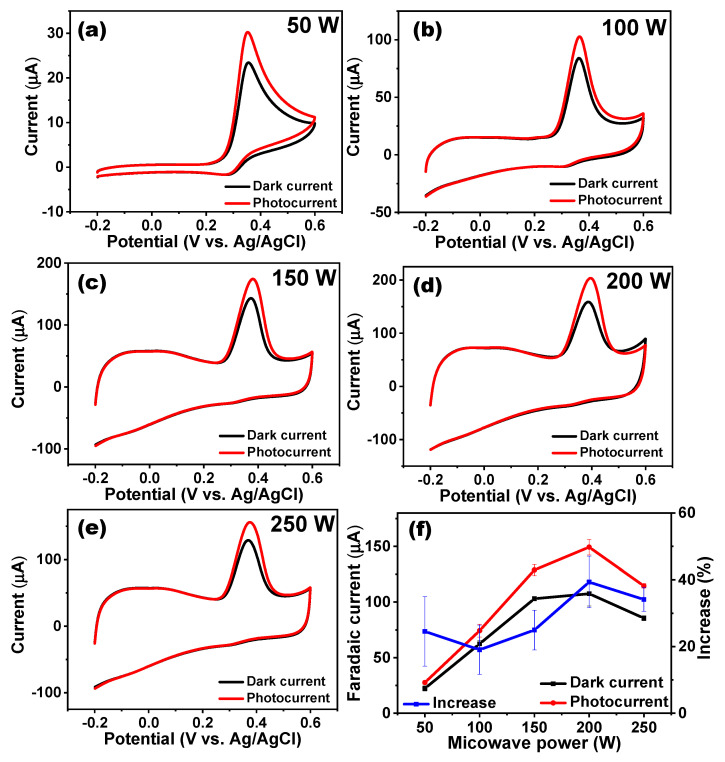
Cyclic voltammograms of (**a**) GONR(50 W), (**b**) GONR(100 W), (**c**) GONR(150 W), (**d**) GONR(200 W), and (**e**) GONR(250 W) with or without light (AM 1.5) illumination. (electrolyte: 0.3 mM UA + 0.1 M PBS, scan rate: 50 mV/s) (**f**) The corresponding summary figure of Faradaic currents from Figure 3a to Figure 3e. The screen-printed carbon electrode was used for preparing the working electrode in Figure 3.

The cyclic voltammetry (CV) curves in Figure 3a–e show the dark currents and photocurrents of UA oxidation, using GONR(50–250 W) on SPCEs before and after AM 1.5 light irradiation. The electrolyte was 0.1 M phosphate-buffered saline (PBS) containing 0.3 mM UA, and the scan rate remained at 50 mV/s. The oxidation peaks of UA were located at 0.35 V to 0.39 V. The increase in Faradaic currents was gradually raised from 34.1% (GONR(50 W)) to 50.0% (GONR(200 W)), and then was dropped to 31.4% (GONR(250 W)). Among all catalysts, GONR(200 W) exhibited both the highest Faradaic dark current as well as the highest photocurrent. Its Faradaic dark current of 97.7 mA could grow up to 146.5 mA under light irradiation, demonstrating that GONR(200 W) was our best sample at this stage. In Figure 3f, using the low dark Faradaic current as the denominator for calculating the increase led to a more extensive error bar than the high dark Faradaic current. Conversely, we summarized the error bars in the increase from the experimental data by repeating the procedure five times. Consequently, they may appear to have had a wider distribution. The CV results for detecting ascorbic acid, dopamine, and UA are displayed in Figure S3 before and after light (AM 1.5) irradiation using GONR(200 W). Unlike UA oxidation, there was no obvious photo-enhanced phenomenon for ascorbic acid and dopamine detection. Therefore, in following experiments, we only focused on the UA oxidation reaction. It is worthwhile to mention that the double-layer CV curves with no UA added are recorded in Figure S4, indicating that GONR(200 W) could have also owned the largest capacitance among all ribbons.

**Figure 4:**
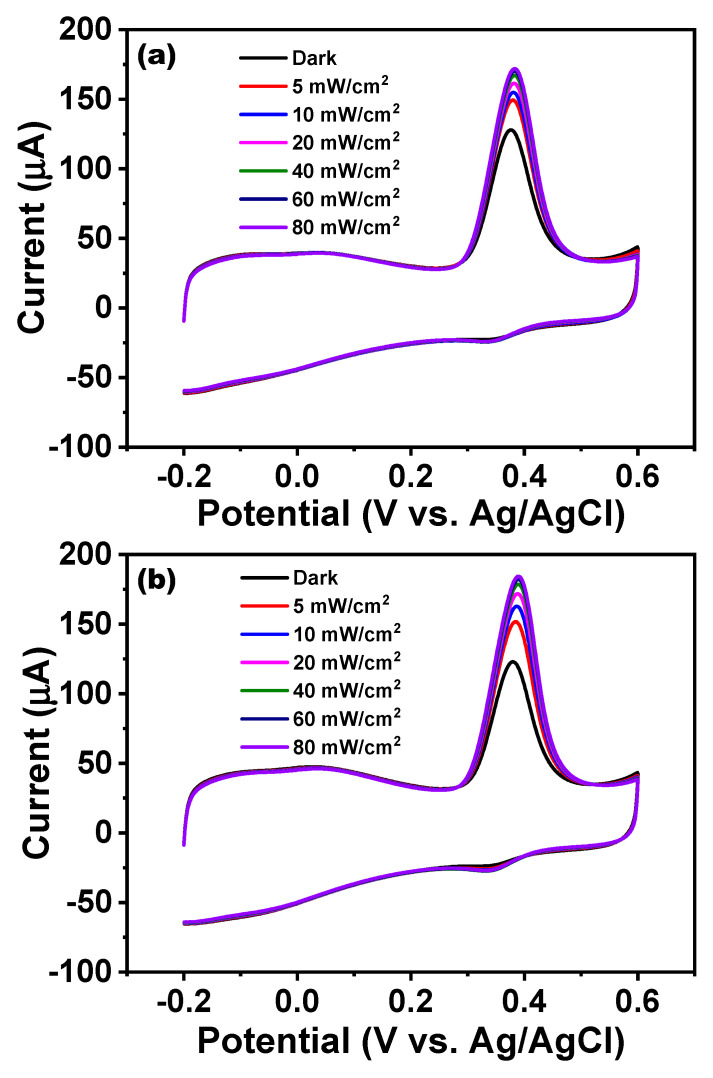
Cyclic voltammograms of GONR(200 W) with or without light (LED) illumination using the wavelength of (**a**) 420 nm and (**b**) 656 nm. (Electrolyte: 0.3 mM UA + 0.1 M PBS, scan rate: 50 mV/s). The light intensity of LED increases from 5 mW/cm^2^ to 80 mW/cm^2^. The glassy carbon electrode was used for preparing the working electrode in Figure 4.

**Figure 5:**
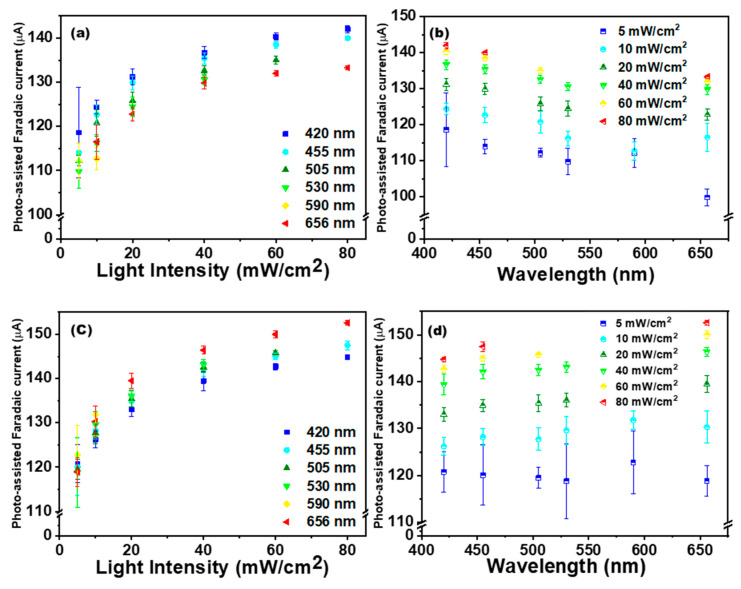
The summary figures of Faradaic currents in cyclic voltammograms of GONR(200 W) with light (LED) illumination using (**a**,**c**) different wavelengths and (**b**,**c**) light intensities. (**a**,**b**) The sequences of wavelength selections switch from 420 nm to 656 nm. (**c**,**d**) The sequences switch from 656 nm to 420 nm.

**Figure 6:**
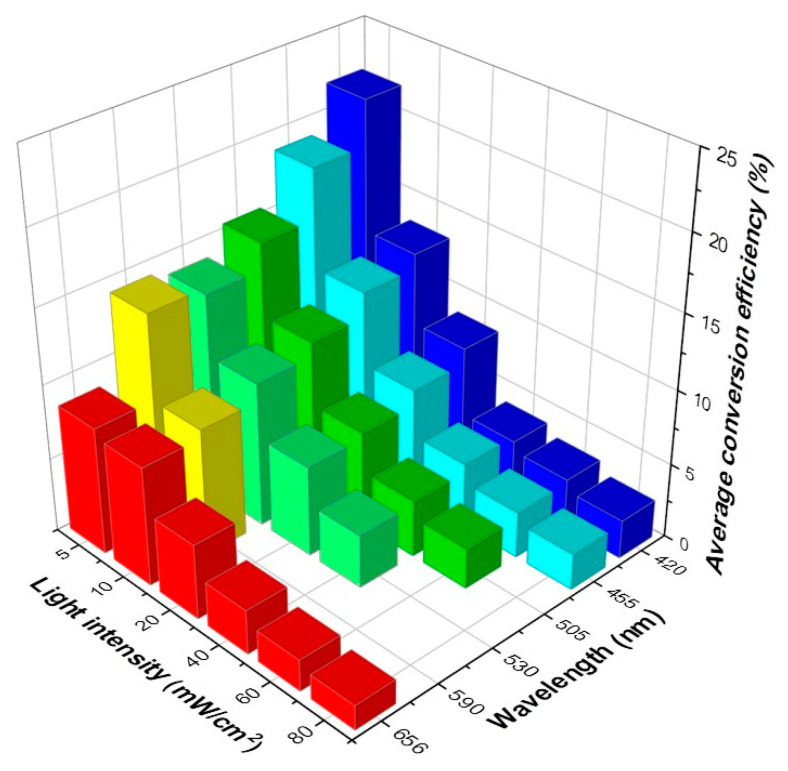
The summary figure of average conversion efficiencies in cyclic voltammograms of GONR(200 W) with light (LED) illumination using different wavelengths and light intensities derived from Figure 5.

Figure 4a shows that the anodic oxidation peak current without light irradiation was 128.1 µA at a potential of approximately 0.38 V. After irradiation with blue light of 420 nm at an intensity of 5 mW/cm^2^ through LED, the anodic current rose to 149.4 µA, and the Faradaic current was increased by approximately 22.7%. Under subsequent blue light irradiations at different intensities of 10, 20, 40, 60, and 80 mW/cm^2^, the Faradaic currents were increased by 28.7%, 35.7%, 41.4%, 45.1%, and 46.9%, respectively. Thus, the Faradaic current increased with the light intensity, but at a gradually decreasing rate. Conversely, Figure 4b displays a CV plot under red light (656 nm) irradiation. At a potential of 0.38 V, the anodic peak current without irradiation was 122.8 µA. After red light irradiation with an intensity of 5 mW/cm^2^, the anodic current rose to 151.93 µA, and the Faradaic current increased by approximately 32.16%. Similarly, the Faradaic currents were increased by 44.8%, 55.1%, 62.7%, 66.7%, and 69.6% under the subsequent red light irradiation at the intensities of 10, 20, 40, 60, and 80 mW/cm^2^. Thus, under both blue and red light irradiation, the GONR(200 W) exhibited significant improvement in photo-assisted electrochemical biosensing. This phenomenon of using red light followed a similar trend observed under blue light irradiation, that is, the Faradaic current increased with the light intensity at a decreasing rate. In order to more carefully investigate this, six wavelengths from an LED were utilized for PEC experiments in addition to the original AM 1.5 light source. Figure 5a,c shows the processed Faradaic current data in CV measurement under light irradiations of 420, 455, 505, 530, 590, and 656 nm at different intensities. For example, when choosing the wavelength of 420 nm, we started the measurements by gradually increasing the light intensities from 5, 10, 20, 40, 60 to 80 mW/cm^2^. After finishing the 420 nm, we moved to the following wavelengths of 455, 505, 530, 590, and 656 nm, one by one. On the contrary, we started from 420 nm to 656 nm in Figure 5a, but reversed the sequences from 656 nm to 420 nm in Figure 5c. Irrespective of the wavelengths of light, we clearly observed that the light-assisted Faradaic current gradually increased with the light intensity, but at a gradually decreasing rate under all six wavelengths. Notably, the increase in the Faradaic current decreased after changing wavelengths, presumably due to the reduced catalyst activity over the extended experimental time. Figure 5b,d shows that the increase in the photo-assisted Faradaic current could be independent of wavelengths. Therefore, this suggests that light intensity had a stronger effect than the wavelength on enhancing the photo-assisted Faradaic current. As seen in Figure 5, we always started the experiments from low light intensity to high intensity for one specific wavelength. The total experiment time for either Figure 5a,b or Figure 5c,d took approximately 7 h. It was observed that the data from the low light intensity of 5 mW/cm^2^ usually had a more expansive error bar. Finally, differential pulse voltammetry (DPV) results from when we changed UA concentrations with or without blue light (80 mW/cm^2^) irradiation are presented in Figure S5. Under a dark circumstance, it was found that the anodic oxidation peak current was barely detectable at relatively low UA concentrations, that is, concentrations below 10 μM. The sensitivity displayed in Figure S5 is the slope divided by the carbon electrode’s geometry area (3 mm diameter circle). The calculated sensitivity was 2.69 µA/µMcm^2^ for the dark current. Figure S5c shows the response current depending on UA concentration under blue light (80 mW/cm^2^) irradiation. The calculated sensitivity was 5.52 µA/µMcm^2^ below 200 mM. It shows a considerably higher sensitivity to low-concentration UA with light irradiation compared to that without light irradiation. A summary on the average conversion efficiencies in cyclic voltammograms of GONR(200 W) with LED irradiation under different wavelengths and light intensities is depicted in Figure 6, with detailed information provided in Figure S6 and Tables S1–S4. There are two examples for calculating the conversion efficiencies displayed in Supplementary Tables S1 and S2. In Supplementary Table S1, we dealt with the case for 420 nm with 5 mW/cm^2^. In Supplementary Table S2, we turned to 656 nm with 80 mW/cm^2^. All the exact numbers can be found in the Supplementary Tables S3 and S4. On the one hand, the photon with the lower wavelength and higher energy could exhibit the better average conversion efficiency. On the other hand, the photon from the lower light intensity at the same wavelength could also own better conversion efficiency. Therefore, we can observe that the best average photon-to-electron conversion efficiency is over 20.00% for the short wavelength of 420 nm and the low intensity of 5 mW/cm^2^.

## 4. Conclusions

In summary, we have applied a microwave heating process to unzip MWCNTs into GONRs in a mixed acid solution. The GONRs were used to modify a working electrode for the photo-assisted biosensing of UA. The GONR(200 W) displayed better photo-enhanced electrochemical UA oxidation activity than other nanoribbons. Furthermore, irradiating the electrode surface with different light sources increased the response current over that obtained in a dark environment. For 0.3 mM UA and a light intensity of 5 mW/cm^2^, the Faradaic current of GONR(200 W) increased by approximately 20.0%, irrespective of light wavelengths. At a light intensity of 80 mW/cm^2^, the Faradaic current was increased by around 40.0%, suggesting that light intensity plays a significant role in our experiments. A DPV experiment showed an approximately two-fold increase in sensitivity from 2.687 µA/µMcm^2^ to 5.517 µA/µMcm^2^ under light irradiation at low concentrations. Therefore, it is believed that GONRs exhibited an excellent photocurrent response under light illumination for PEC biosensing applications.

## Figures and Tables

**Table 1:** Summary of PEC biosensors that use a variety of materials and light sources.

Material	Electrode	Analyte	Light Source	Method	Ref.
TiO_2_/g-C_3_N_4_ w dendrimer, and alkaline phosphatase	ITO	Protein Kinase A	N/A	I-t	[9]
CQDs *^a^*-functionalized MnO_2_ NSs *^b^* with GO_x_-labeled AFB_1_-bovine serum albumin	FTO	Aflatoxin B_1_	500 W Xe lamp with a 420 nm cutoff filter	I-t	[10]
rGO *^c^*/BiFeO_3_	FTO	Prostate-specific antigen	LED light (10 W)	I-t	[12]
CdS–In_2_S_3_	ITO	Bleomycin	Xe lamp (λ > 420 nm)	I-t	[13]
PbS QDs *^d^*/Co_3_O_4_/Au NPs *^e^* /BiOI NSs *^c^*/B-TiO_2_ NPs *^e^*	ITO	Procalcitonin	N/A	I-t	[14]
PtNi NWs *^f^*/BR-CN *^g^*	FTO	Chloramphenicol	300 W Xe lamp (λ > 420 nm)	I-t	[15]
PDANS *^h^*-CdS QDs *^d^*/Ag //ERGO*^i^*-TiO_2_	ITO	Prostate specific antigen	LED (20 mW/cm^2^)	I-t	[16]
CDs *^j^*-GO *^k^*/Au–CuO–Cu_2_O	GCE	Progesterone	Xe lamp (λ > 420 nm)	I-t	[17]
PDDA *^l^/*graphene/TiO_2_	Gold on SiO_2_/Si	Hexavalent chromium	150 W Xe lamp	I-t, CV	[18]
Bi^3+^/B-TiO_2_/rGO *^b^*	ITO	Tobramycin	250 W Xe lamp (λ > 400 nm)	I-t	[19]
Ag NCs *^m^*/Ag NPs *^e^*/GO	Gold	Dopamine and glutathione	LED (365 nm, 190 mW)	I-v, CV	[20]
GONR *^n^*	GCE	Uric acid	LED (6 intensities as well as 6 wavelengths)	CV, DPV	This work

*^a^* CQDs: carbon quantum dots. *^b^* rGO: reduced graphene oxide. *^c^* NSs: nanosheets. *^d^* QDs: quantum dots. *^e^* NPs: nanoparticles. *^f^* NWs: nanowires. *^g^* BR-CN: benzene-ring doped g-C_3_N_4_. *^h^* PDANS: quinone-rich polydopamine nanospheres. *^i^* ERGO: electrochemically reduced graphene oxide. *^j^* CDs: carbon dots. *^k^* GO: graphene oxide. *^l^* PDDA: poly(diallyldiamine chloride). *^m^* NCs: nanoclusters. *^n^* GONR: graphene oxide nanoribbon.

## Supplementary Materials

The following are available online at www.mdpi.com/2079-4991/11/10/2693/s1, Figure S1: Raman spectra of MWCNT and GONRs, Figure S2: XPS bonding ratios of MWCNT and GONRs. Figure S3: CVs of GONR(200 W)., Figure S4: CVs of GONRs. Figure S5: DPVs of GONR(200 W). Figure S6: Conversion efficiencies of GONR(200 W), Table S1: Conversion efficiency (%) calculation for 420 nm with 5 mW/cm^2^, Table S2: Conversion efficiency (%) calculation for 656 nm with 80 mW/cm^2^, Table S3: Calculation details when light illumination was carried out from 420 nm to 656 nm, Table S4: Calculation details when light illumination was carried out from 656 nm to 420 nm.
