# Supplementary material for: Visible-Light-Assisted Photoelectrochemical Biosensing of Uric Acid Using Metal-Free Graphene Oxide Nanoribbons
Source: Nanomaterials (Basel). 2021 Oct 13;11(10):2693. doi: 10.3390/nano11102693 (PMC8538689; doi:10.3390/nano11102693)
Supplement: Supplementary file 1 [file nanomaterials-11-02693-s001.zip › nanomaterials-1410212-supplementary.pdf]

nanomaterials

Supplementary Materials

Visible-Light-Assisted Photoelectrochemical Biosensing of Uric Acid Using Metal-Free Graphene Oxide Nanoribbons

Chia-Liang Sun [1,2,*], Cheng-Hsuan Lin [1], Chia-Heng Kuo [1], Chia-Wei Huang [1], Duc Dung Nguyen [3], Tsu-Chin Chou [4], Cheng-Ying Chen [5], Yu-Jen Lu [2,*]

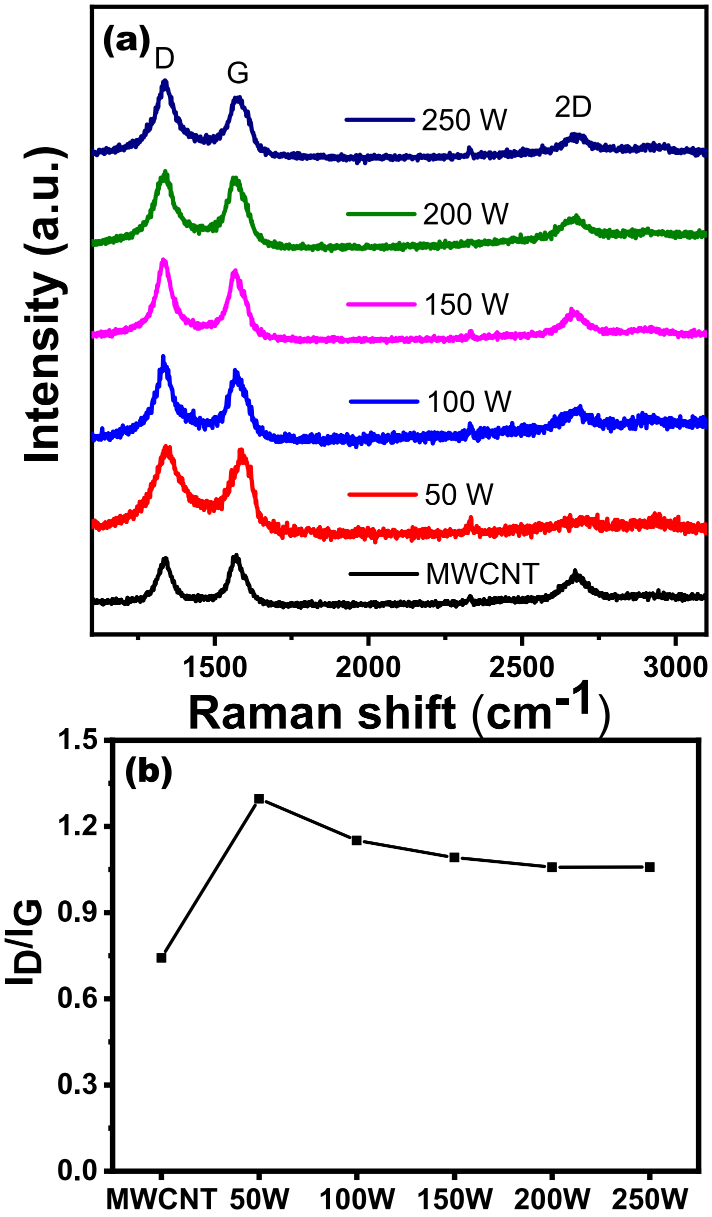

Figure S1. (**a**) Raman spectra of MWCNT, GONR(50 W), GONR(100 W), GONR (150 W), GONR(200 W), and GONR (250 W) before and after unzipping process. (**b**) The corresponding I_D/I_G summary figure of Figure S1(a).

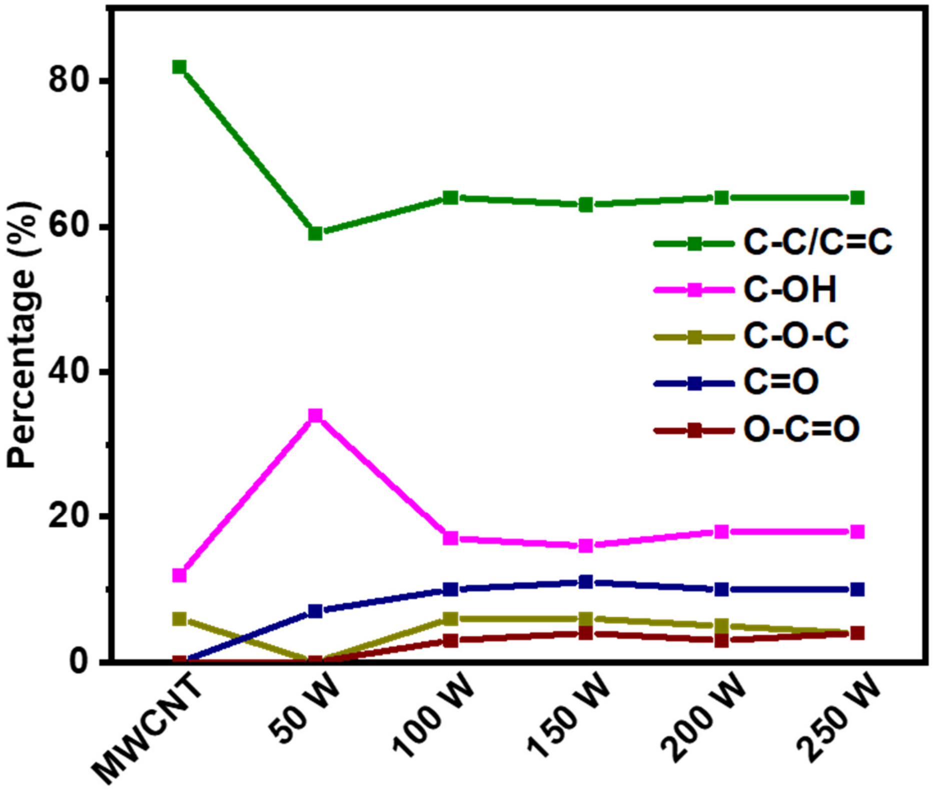

Figure S2. The corresponding bonding ratios of MWCNT, GONR(50 W), GONR(100 W), GONR(150 W), GONR(200 W), and GONR(250 W) derived from Figure 2.

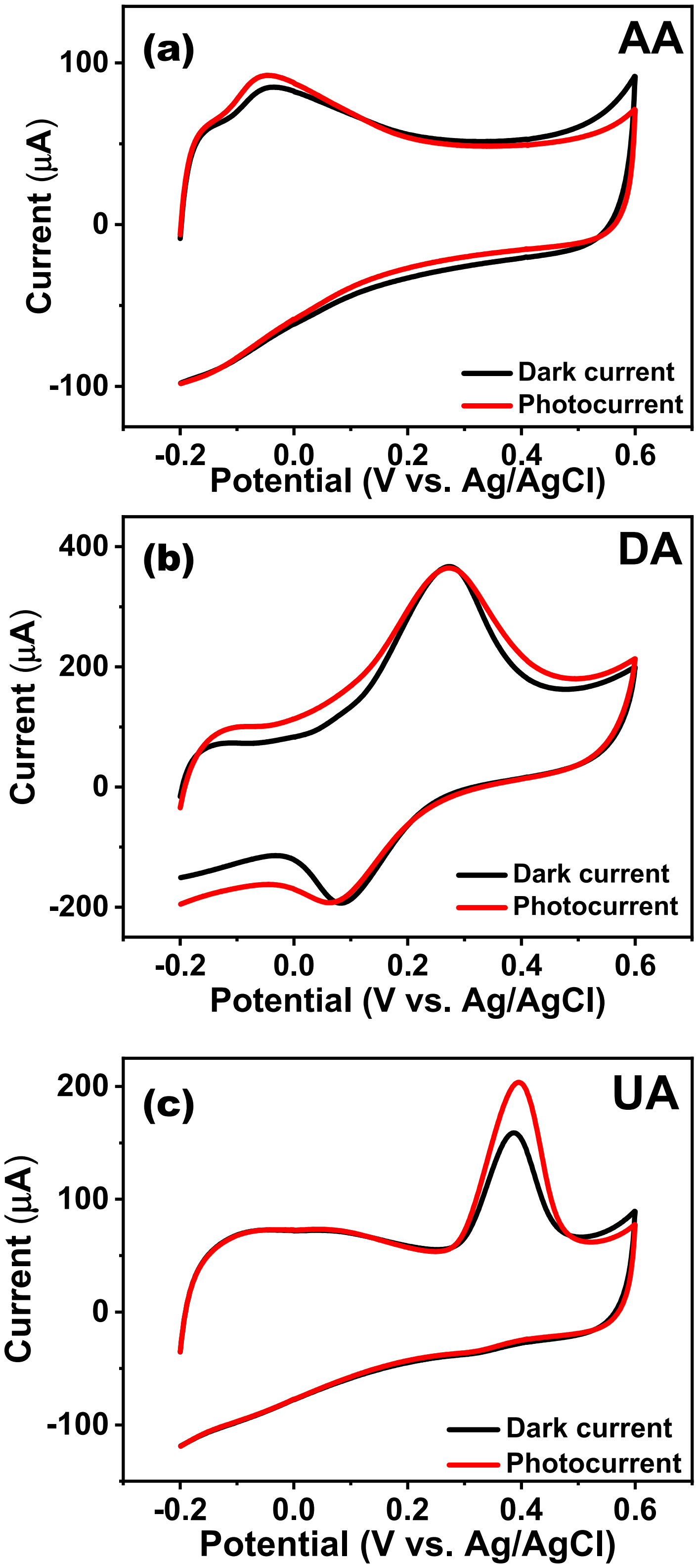

Figure S3. Cyclic voltammograms of GONR(200 W) in the electrolyte containing 0.1 M PBS and (**a**) 1 mM AA, (**b**) 1 mM DA, and (**c**) 0.3 mM UA at the scan rate of 50 mV/s with or without light (AM 1.5) illumination. The screen-printed carbon electrode was used for preparing the working electrode in Figure S3.

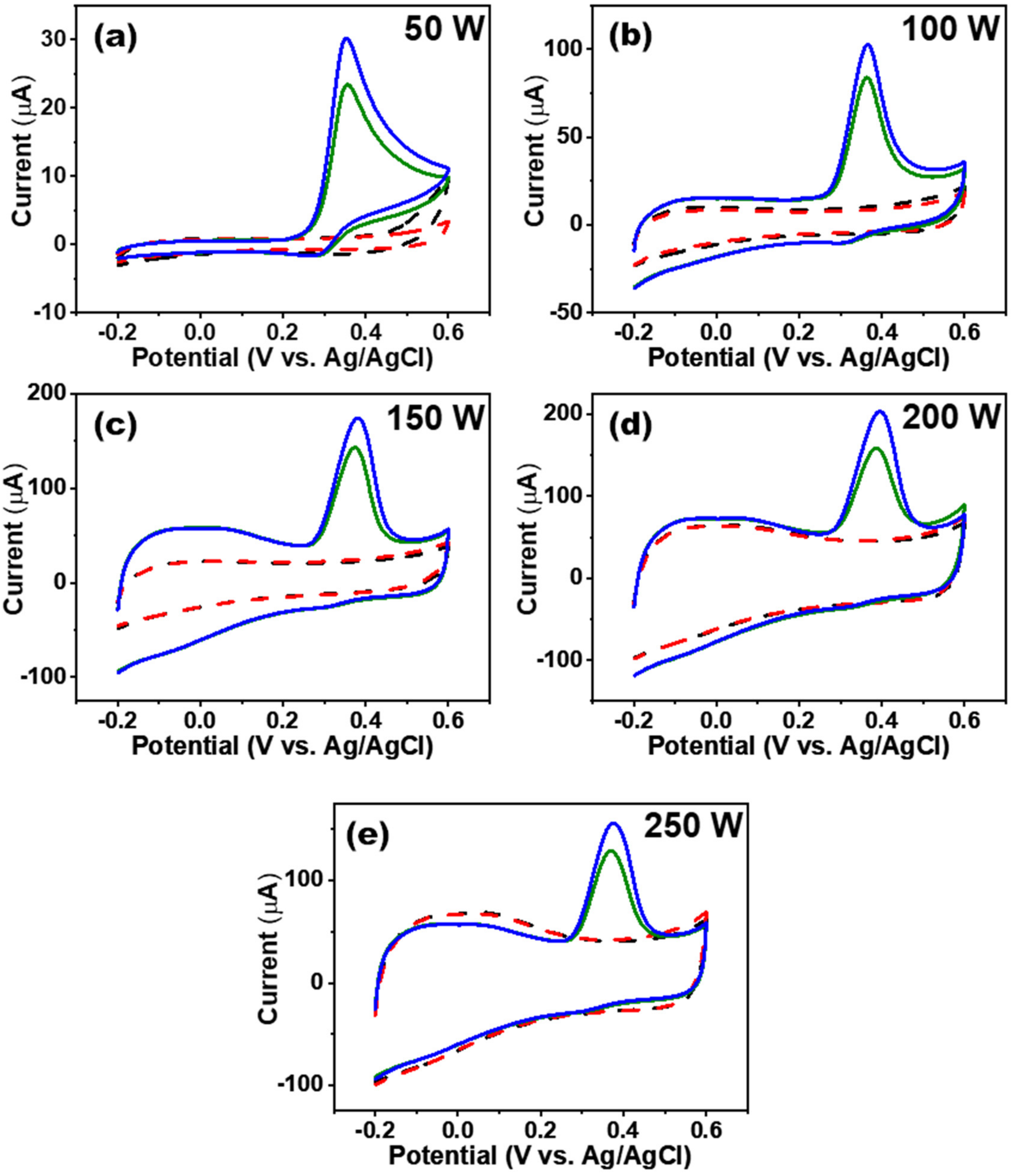

Figure S4. Cyclic voltammograms of (**a**) GONR(50 W), (**b**) GONR(100 W), (**c**) GONR(150 W), (**d**) GONR(200 W), and (**e**) GONR(250 W) with (blue line) or without (green line) light (AM 1.5) illumination. (electrolyte: 0.3 mM UA + 0.1 M PBS, scan rate: 50 mV/s) The dash lines are the curves for dark currents with 0 mM UA with (black) or without (red) illumination. The screen-printed carbon electrode was used for preparing the working electrode in Figure S4.

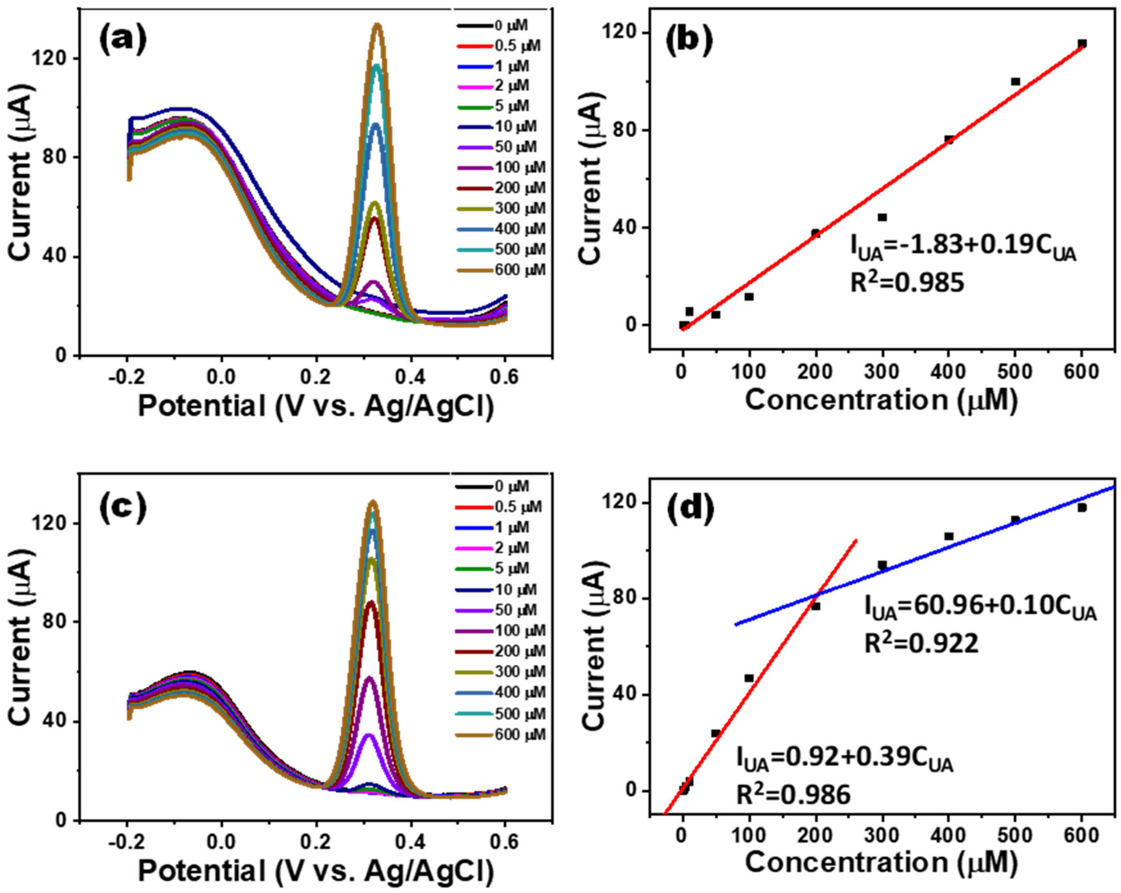

Figure S5 Differential pulse voltammetry curves of GONR(200 W) in 0.1 M PBS containing 0.5–600.0 mM UA (**a**) without light illumination and (**c**) with LED light (420 nm, 80 mW/cm^2). (**b, d**) The corresponding plots of the oxidation current of UA(I_{UA}) vs. the UA concentration (C_{UA}) for (a, c). The glassy carbon electrode was used for preparing the working electrode in Figure S5.

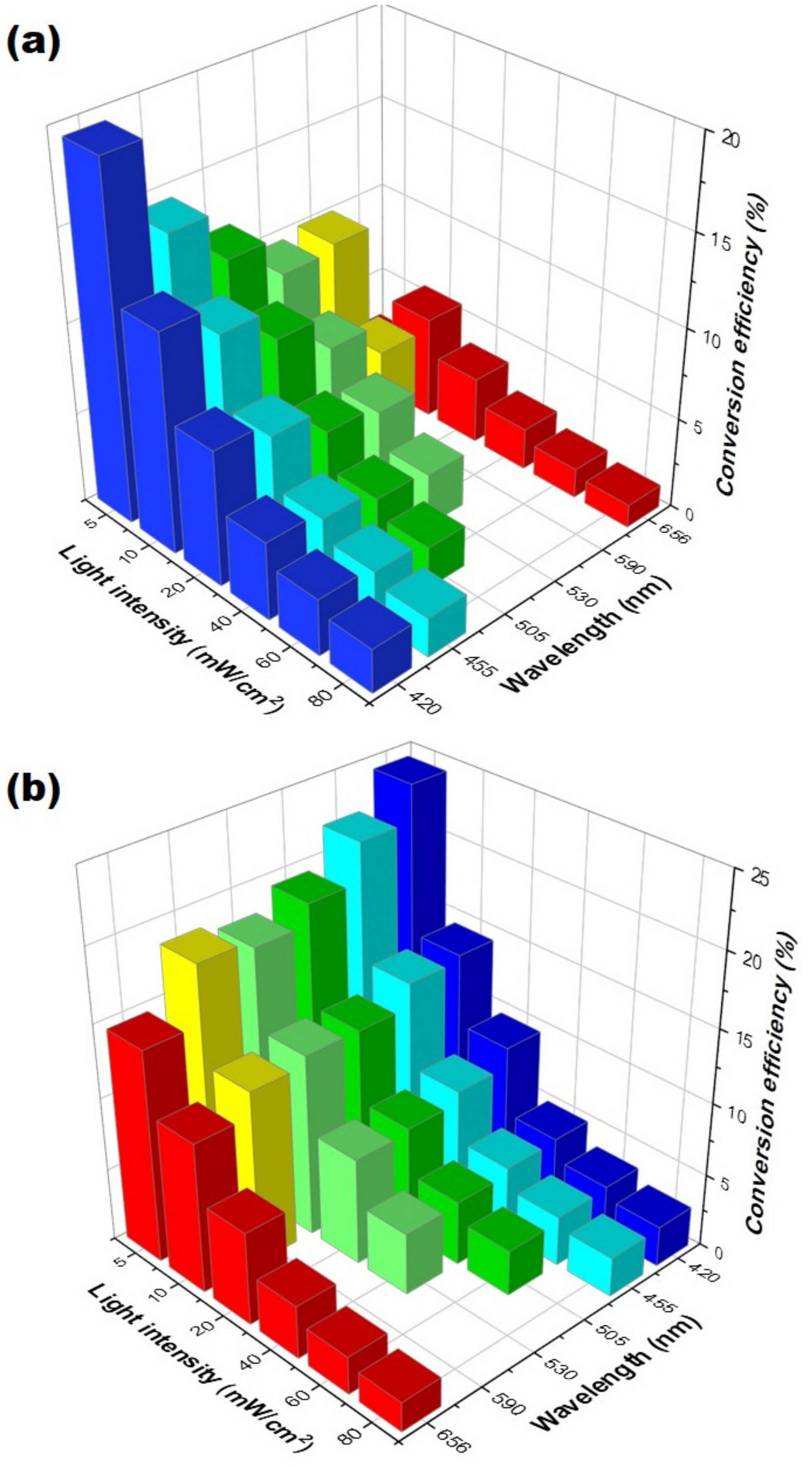

Figure S6. The summary figure of conversion efficiencies in cyclic voltammograms of GONR(200 W) with light (LED) illumination using different wavelengths and light intensities derived from Figure 5. (**a**) The sequences of wavelength selections switch from 420 nm to 656 nm. (**b**) The sequences switch from 656 nm to 420 nm.

Table S1. Conversion efficiency (%) calculation for 420 nm with 5 mW/cm[2.]

The cyclic voltammetry (CV) measurement results with an anodic peak for oxidation of uric acid

$$\text{Conversion Efficiency} = \frac{\text{Number of electrons per Time interval}}{\text{Number of photons per Time interval}} \times 100$$

$$= \frac{(\text{Photo assisted } - \text{Dark Faradaic current}) \times 10^6 \times 6.242 \text{ x } 10^{18} \times \text{Time interval}}{\text{Photon flux x Area of 3 mm} - \text{diameter circle x Time interval}} \times 100$$

Light Source	LED Lamp
Light intensity (mW/cm^2)	5
Wavelength (nm)	420
Photon Flux<br>(number of photons/sec˙m^2)	$5 \times 10 \times 420 \times 10^{16}/(1.24 \times 1.6)$<br>$= 1.06 \times 10^{20}$
Area of 3-mm-diameter circle (m^2)	7.1×10^{-6}
Scan rate (V/s)	0.05
Peak potential step (V)	0.001
Time interval (s)	0.001/0.05 = 0.02
Number of photons<br>(0.02 sec and 3-mm-diameter circle)	$1.06 \times 10^{20} \times 7.1 \times 10^{-6} \times 0.02 = 1.5 \times 10^{13}$
Dark Faradaic current (μA)	95.19
Photo-assisted Faradaic current (μA)	118.6
Number of electrons per Coulomb	6.242×10^{18}
0.02 sec (number of electrons)	$(118.6 - 95.19) \times 6.242 \times 10^{12} \times 0.02$<br>$= 2.92 \times 10^{12}$
Conversion efficiency at peak in CV	$2.92 \times 10^{12} / 1.5 \times 10^{13} = 19.44$

Table S2. Conversion efficiency (%) calculation for 656 nm with 80 mW/cm$^{2.}$

Light Source	LED Lamp
Light intensity (mW/cm^2)	80
Wavelength (nm)	656
Photon Flux<br>(number of photons/sec˙m^2)	80 × 10 × 656 × 10^{16}/(1.24 × 1.6)<br>= 2.65 × 10^{21}
Area of 3-mm-diameter circle (m^2)	7.1 × 10^{-6}
Scan rate (V/s)	0.05
Peak potential step (V)	0.001
Time interval (s)	0.001/0.05 = 0.02
Number of photons<br>(0.02 sec and 3-mm-diameter circle)	2.65 × 10^{21} × 7.1 × 10^{-6} × 0.02 = 3.76 × 10^{14}
Dark Faradaic current (μA)	91.46
Photo-assisted Faradaic current (μA)	152.6
Number of electrons per Coulomb	6.242 × 10^{18}
0.02 sec (number of electrons)	(152.6 − 91.46) × 6.242 × 10^{12} × 0.02<br>= 7.63 × 10^{12}
Conversion efficiency at peak in CV	7.63 × 10^{12}/3.76 × 10^{14} = 2.03

Table S3. Calculation details when light illumination was carried out from 420 nm to 656 nm.

Wavelength (nm)	Light intensity (mW/cm^2)	Photon flux (# of photons/sec × m^2)	# of photons (0.02 sec and 3-mm-diameter circle)	Dark Faradaic current (µA)	Photo-assisted Faradaic current (µA)	0.02 sec (# of electrons)	Conversion Efficiency (%) at peak in CV
420	80	1.69×10^{21}	2.40×10^{14}	95.19	142	5.84×10^{12}	2.43
420	60	1.27×10^{21}	1.80×10^{14}	95.19	140.3	5.63×10^{12}	3.12
420	40	8.47×10^{20}	1.20×10^{14}	95.19	136.7	5.18×10^{12}	4.31
420	20	4.23×10^{20}	6.01×10^{13}	95.19	131.2	4.50×10^{12}	7.48
420	10	2.12×10^{20}	3.01×10^{13}	95.19	124.4	3.65×10^{12}	12.13
420	5	1.06×10^{20}	1.50×10^{13}	95.19	118.6	2.92×10^{12}	19.44
455	80	1.83×10^{21}	2.61×10^{14}	95.19	140	5.59×10^{12}	2.15
455	60	1.38×10^{21}	1.95×10^{14}	95.19	138.5	5.41×10^{12}	2.77
455	40	9.17×10^{20}	1.30×10^{14}	95.19	135.4	5.02×10^{12}	3.85
455	20	4.59×10^{20}	6.51×10^{13}	95.19	129.9	4.33×10^{12}	6.65
455	10	2.29×10^{20}	3.26×10^{13}	95.19	122.7	3.43×10^{12}	10.55
455	5	1.15×10^{20}	1.63×10^{13}	95.19	114	2.35×10^{12}	14.42
505	80	2.04×10^{21}	2.89×10^{14}	95.19	-	-	-
505	60	1.53×10^{21}	2.17×10^{14}	95.19	135	4.97×10^{12}	2.29
505	40	1.02×10^{21}	1.45×10^{14}	95.19	132.6	4.67×10^{12}	3.23
505	20	5.09×10^{20}	7.23×10^{13}	95.19	125.8	3.82×10^{12}	5.29
505	10	2.55×10^{20}	3.61×10^{13}	95.19	120.8	3.20×10^{12}	8.85
505	5	1.27×10^{20}	1.81×10^{13}	95.19	112.2	2.12×10^{12}	11.75
530	80	2.14×10^{21}	3.03×10^{14}	95.19	-	-	-
530	60	1.60×10^{21}	2.28×10^{14}	95.19	-	-	-
530	40	1.07×10^{21}	1.52×10^{14}	95.19	130.6	4.42×10^{12}	2.91
530	20	5.34×10^{20}	7.59×10^{13}	95.19	124.5	3.66×10^{12}	4.82
530	10	2.67×10^{20}	3.79×10^{13}	95.19	116.2	2.62×10^{12}	6.91
530	5	1.34×10^{20}	1.90×10^{13}	95.19	109.8	1.82×10^{12}	9.62
590	80	2.38×10^{21}	3.38×10^{14}	95.19	-	-	-
590	60	1.78×10^{21}	2.53×10^{14}	95.19	-	-	-
590	40	1.19×10^{21}	1.69×10^{14}	95.19	-	-	-
590	20	5.95×10^{20}	8.45×10^{13}	95.19	-	-	-
590	10	2.97×10^{20}	4.22×10^{13}	95.19	112.7	2.19×10^{12}	5.18
590	5	1.49×10^{20}	2.11×10^{13}	95.19	112.1	2.11×10^{12}	10.00
656	80	2.65×10^{21}	3.76×10^{14}	95.19	133.3	4.76×10^{12}	1.27
656	60	1.98×10^{21}	2.82×10^{14}	95.19	132	4.60×10^{12}	1.63
656	40	1.32×10^{21}	1.88×10^{14}	95.19	129.8	4.32×10^{12}	2.30
656	20	6.61×10^{20}	9.39×10^{13}	95.19	122.8	3.45×10^{12}	3.67
656	10	3.31×10^{20}	4.70×10^{13}	95.19	116.5	2.66×10^{12}	5.67
656	5	1.65×10^{20}	2.35×10^{13}	95.19	99.9	5.88×10^{11}	2.50

Table S4. Calculation details when light illumination was carried out from 656 nm to 420 nm.

Wavelength (nm)	Light intensity (mW/cm^2)	Photon flux (# of photons/sec × m^2)	# of photons (0.02 sec and 3-mm-diameter circle)	Dark Faradaic current (μA)	Photo-assisted Faradaic current (μA)	0.02 sec (# of electrons)	Conversion Efficiency (%) at peak in CV
420	80	1.69×10^{21}	2.40×10^{14}	91.46	144.8	6.66×10^{12}	2.77
420	60	1.27×10^{21}	1.80×10^{14}	91.46	142.7	6.40×10^{12}	3.55
420	40	8.47×10^{20}	1.20×10^{14}	91.46	139.4	5.98×10^{12}	4.98
420	20	4.23×10^{20}	6.01×10^{13}	91.46	133	5.19×10^{12}	8.63
420	10	2.12×10^{20}	3.01×10^{13}	91.46	126.2	4.34×10^{12}	14.43
420	5	1.06×10^{20}	1.50×10^{13}	91.46	120.8	3.66×10^{12}	24.37
455	80	1.83×10^{21}	2.61×10^{14}	91.46	147.5	7.00×10^{12}	2.69
455	60	1.38×10^{21}	1.95×10^{14}	91.46	145	6.68×10^{12}	3.42
455	40	9.17×10^{20}	1.30×10^{14}	91.46	142.1	6.32×10^{12}	4.85
455	20	4.59×10^{20}	6.51×10^{13}	91.46	134.9	5.42×10^{12}	8.33
455	10	2.29×10^{20}	3.26×10^{13}	91.46	128.2	4.59×10^{12}	14.08
455	5	1.15×10^{20}	1.63×10^{13}	91.46	120.1	3.58×10^{12}	21.96
505	80	2.04×10^{21}	2.89×10^{14}	91.46	-	-	-
505	60	1.53×10^{21}	2.17×10^{14}	91.46	145.8	6.78×10^{12}	3.13
505	40	1.02×10^{21}	1.45×10^{14}	91.46	142.5	6.37×10^{12}	4.41
505	20	5.09×10^{20}	7.23×10^{13}	91.46	135.4	5.49×10^{12}	7.59
505	10	2.55×10^{20}	3.61×10^{13}	91.46	127.7	4.52×10^{12}	12.52
505	5	1.27×10^{20}	1.81×10^{13}	91.46	119.5	3.50×10^{12}	19.37
530	80	2.14×10^{21}	3.03×10^{14}	91.46	-	-	-
530	60	1.60×10^{21}	2.28×10^{14}	91.46	-	-	-
530	40	1.07×10^{21}	1.52×10^{14}	91.46	143.1	6.45×10^{12}	4.25
530	20	5.34×10^{20}	7.59×10^{13}	91.46	136	5.56×10^{12}	7.33
530	10	2.67×10^{20}	3.79×10^{13}	91.46	129.6	4.76×10^{12}	12.55
530	5	1.34×10^{20}	1.90×10^{13}	91.46	118.8	3.41×10^{12}	18.00
590	80	2.38×10^{21}	3.38×10^{14}	91.46	-	-	-
590	60	1.78×10^{21}	2.53×10^{14}	91.46	-	-	-
590	40	1.19×10^{21}	1.69×10^{14}	91.46	-	-	-
590	20	5.95×10^{20}	8.45×10^{13}	91.46	-	-	-
590	10	2.97×10^{20}	4.22×10^{13}	91.46	131.8	5.04×10^{12}	11.93
590	5	1.49×10^{20}	2.11×10^{13}	91.46	122.8	3.91×10^{12}	18.53
656	80	2.65×10^{21}	3.76×10^{14}	91.46	152.6	7.63×10^{12}	2.03
656	60	1.98×10^{21}	2.82×10^{14}	91.46	150	7.31×10^{12}	2.59
656	40	1.32×10^{21}	1.88×10^{14}	91.46	146.4	6.86×10^{12}	3.65
656	20	6.61×10^{20}	9.39×10^{13}	91.46	139.5	6.00×10^{12}	6.39
656	10	3.31×10^{20}	4.70×10^{13}	91.46	130.3	4.85×10^{12}	10.33
656	5	1.65×10^{20}	2.34×10^{13}	91.46	118.9	3.43×10^{12}	14.61
